# Ignorance is not bliss: evolutionary naiveté in an endangered desert fish and implications for conservation

**DOI:** 10.1098/rspb.2022.0752

**Published:** 2022-08-31

**Authors:** Craig A. Stockwell, Madison R. Schmelzer, Bailey E. Gillis, Cody M. Anderson, Brian D. Wisenden

**Affiliations:** ^1^ Department of Biological Sciences, Environmental and Conservation Sciences Program, North Dakota State University, PO Box 6050, Fargo, ND 58108, USA; ^2^ Biosciences Department, Minnesota State University Moorhead, 1104 7th Avenue South, Moorhead, MN 56563, USA

**Keywords:** predator naiveté, insular populations, desert fishes, invasive species, novel predators, chemical alarm cues

## Abstract

Predator naiveté has been invoked to explain the impacts of non-native predators on isolated populations that evolved with limited predation. Such impacts have been repeatedly observed for the endangered Pahrump poolfish, *Empetrichthys latos*, a desert fish species that evolved in isolation since the end of the Pleistocene. We tested Pahrump poolfish anti-predator responses to conspecific chemical alarm cues released from damaged epidermal tissue in terms of fish activity and water column position. Pahrump poolfish behavioural responses to conspecific alarm cues did not differ from responses to a dechlorinated tap water control. As a positive control, the well-studied fathead minnow, *Pimephales promelas*, showed significant alarm cue responses in terms of reduced activity and lowered water column position. The density of epidermal club cells, the presumptive source of alarm cues, was significantly lower in Pahrump poolfish relative to fathead minnows. Therefore, anti-predator competence mediated by conspecific alarm cues does not seem to be a component of the ecology of Pahrump poolfish. These findings provide a proximate mechanism for the vulnerability of Pahrump poolfish to non-native predators, with implications for the conservation and management of insular species.

## 1. Background

A lack of anti-predator traits is broadly observed in populations that evolved under limited predation pressure [1–3]. Such limited predation pressure often occurs on islands [1], areas where natural barriers limit the movement of predators [4], as well as habitats where predators were historically eliminated [5]. The absence of anti-predator behaviours, which is broadly referred to as predator naiveté, can be revealed when prey species encounter non-native predators [1,2]. This is especially the case with non-native predators that have unfamiliar morphology and/or use novel hunting behaviour [2]. Anton *et al*. [3] evaluated ecological and biogeographical correlates of predator naiveté and found it was common among insular vertebrate populations and particularly common in fishes.

In aquatic systems, predator avoidance is often mediated by chemical cues of predators as well as chemical alarm cues released when the skin of conspecifics is damaged during predatory attack [6,7]. Specifically, club cells in the epidermis have been hypothesized to contribute to alarm cues [8,9]. Examples of behavioural responses to alarm cues include decreased activity, increased use of shelter, shoaling and area avoidance [10,11]. Detection and response to chemical alarm cues allow individuals to reduce predation risk [12,13]. Thus, the absence of these behavioural traits could have important implications for the conservation and management of insular species that evolved with low predation pressure.

Predator naiveté may have played a role in the wide-spread decline of desert fishes in the southwestern United States, which evolved in isolated habitats with few fish predators since the recession of the Pleistocene Lakes around 10 000 years ago [14–16]. For example, the arrival of non-native species has been associated with the extinction of both the Monkey Springs pupfish (*Cyprinodon arcuatus*) and the Ash Meadows poolfish (*Empetrichthys merriami*) [16]. Furthermore, non-native species have been associated with the decline of numerous desert fishes [14–17]. Finally, experimental work has shown detrimental impacts of non-native species on several fishes of conservation concern [18–24].

Our work focused on the Pahrump poolfish (*E. latos*) because invasive species are a critical threat to the recovery of this federally listed endangered species [22]. Pahrump poolfish, which evolved in isolation from other fish species since the desiccation of Pleistocene Lake Pahrump approximately 10 000 years ago [25], is the last extant member of its genus. This species no longer occurs in its native habitat and has been managed among numerous refuge habitats since the 1970s [26]. Invasive species have been linked to three events where refuge populations rapidly collapsed [22] (Kevin Guadalupe, Nevada Department of Wildlife, pers. comm.). Further, a series of mesocosm experiments showed that larval production of Pahrump poolfish was eliminated in the presence of western mosquitofish, *Gambusia affinis*, and adult survival of Pahrump poolfish was significantly reduced in the presence of invasive red swamp crayfish, *Procambarus clarkii* [23].

Observations of non-native predator impacts on Pahrump poolfish were consistent with the predator naiveté hypothesis, but the underlying mechanisms have not been evaluated. Understanding such mechanisms would be useful to inform management decisions, especially since efforts to eradicate invasive species have had exceptionally low success rates [27,28]. This study focused on behavioural responses to conspecific alarm cue as well as epidermal histology of Pahrump poolfish. We evaluated responses of Pahrump poolfish to conspecific alarm cue using a standard behavioural assay [29]. We also estimated the densities of epidermal club cells in Pahrump poolfish. As a positive control, we report behavioural responses to alarm cues and epidermal club cell densities for the well-studied fathead minnow (*Pimephales promelas*).

## 2. Methods

Pahrump poolfish were obtained from two of the three extant refuge populations: Spring Mountain Ranch near Las Vegas, NV, USA (36°04′16.9″ N; 115°27′13.7″ W) in 2014 and Shoshone Ponds in South Spring Valley near Ely, NV, USA (38°56'21.8″ N; 114°25'04.6″ W) in 2017. Behavioural assays were conducted during spring 2017, using descendants of fish from the 2014 sample (F1–F3). Histological analyses were based on 29 Pahrump poolfish fish from the 2014 and 2017 collections. These two collections were previously mixed together to provide sufficient sample sizes for mesocosm experiment conducted during the summer of 2017 [23,24]. Laboratory-reared fathead minnows were acquired from EMR Inc., a commercial supplier of research-grade animals subcontracted with the US Environmental Protection Agency, Duluth, MN.

### (a) Behavioural trials

We conducted behavioural trials with laboratory-reared Pahrump poolfish and recorded activity and vertical position of Pahrump poolfish before and after the introduction of a stimulus, either conspecific alarm cue or dechlorinated tap water as a control. Alarm cue was produced by euthanizing individual fish in a solution of 500 mg l^−1^ of tricaine methanesulfonate (MS-222) [30] and filleting skin from both sides of the carcass. Fillets were laid flat on a piece of wet glass to measure skin area before transfer to a beaker of 50 ml dechlorinated tap water resting on crushed ice. For each species, the combined skin from all individuals was homogenized with a hand blender for 30 s and further diluted with dechlorinated tap water to a final concentration of 1.0 cm^2^ skin in 10 ml of water. Previous work with fathead minnows has shown that 1.0 cm^2^ of skin activates 58 000 l of water [10,11]. Thus, this amount of skin extract concentrate (1.0 cm^2^/37.85 l) should illicit a strong behavioural response in both species. Control cue was prepared from dechlorinated tap water. Both alarm and control cue solutions were aliquoted into 10 ml replicates and frozen at −18°C until needed.

Behavioural trials were conducted on single subjects that were individually placed in 37.85 l glass aquaria (electronic supplementary material, figure S1) under broad-spectrum fluorescent lights and maintained on a photoperiod of 12 h light : 12 h dark and maintained at room temperature (approx. 25°C). Each tank received oxygen pumped through an air-powered sponge filter with an additional 2.5 m length of airline tubing inserted into the lift tube of the filter through which test cues could be introduced surreptitiously. A grid of 5 × 5 cm cells was drawn on the outside of the front-facing panel of each test tank. For Pahrump poolfish trials, the large pane of the aquarium faced the viewer, while for fathead trials, the small pane of the aquarium faced the viewer. Test fish were acclimated for 24 h to their experimental tank and randomly assigned to either alarm cue or control treatment. Experimental fish were fed Tetra-min flake food 60–75 min before trials began. For each trial, 50 ml of tank water was withdrawn through the delivery tube with a 60 ml syringe and discarded to rinse any residues from the delivery tube. An additional 50 ml of tank water was drawn and retained to be used later to flush test stimuli from the delivery tube into the tank.

Vertical distribution was determined as the horizontal row in the grid occupied by the test fish every 15 s for Pahrump poolfish or 10 s for fathead minnows, averaged over each 5 min observational period. For both species, the activity of individual fish was measured as the number of lines crossed over 5 min, using the fish's eye to determine its position. Once the pre-stimulus observations were completed, either conspecific chemical alarm cue or dechlorinated tap water (control) was introduced into the tank through the delivery tube, followed by 50 ml flush of previously retained tank water. Immediately after injection of the test stimulus, we recorded fish activity and vertical position for a 5-min post-stimulus observation period.

For both species, vertical position was recorded by two observers in real time. Activity was recorded in real time for trials with fathead minnows; however, activity for trials with Pahrump poolfish was scored from videos recorded with a Canon camcorder (model VIZIA HF R700) placed 1.0–1.5 m directly across from each test tank. Observer effects were minimized by turning off ceiling room lights so that the only illumination came from lights above the test tanks. Observers were positioned 1.5 m away from the tank on an elevated shelf so that observers were not looming above test subjects. Observers moved slowly, calmly and spoke in hushed tones, and the fish were habituated to the presence of people in the laboratory. For both species, experimental tanks were drained, rinsed and refilled with fresh water, and cue injection tubes were replaced after each trial.

For Pahrump poolfish, we tested 84 fish (42 control water and 42 alarm cue). In one trial, the fish displayed unusual swimming movements and in another 25 trials fish had either very low pre-stimulus activity (less than 50 lines, *n* = 22) or very high activity (greater than 400 lines, *n* = 3). In such cases, responses during the post-stimulus period would be inherently limited to a one-sided response (i.e. speeding up for the slow fish, and slowing down for the fast fish). We ran analyses both with the full dataset and with a reduced dataset of 58 fish (29 control trials and 29 alarm cue trials) limited to fish with pre-stimulus movement in the range of 50–400 lines. There was no difference in the outcomes from the two analysis sets, and thus we report the analyses based on the reduced dataset (the full and reduced datasets are publically available in a Dryad Digital Repository [31]). For fathead minnows, we ran 30 trials (15 control and 15 treatment), all of which met the activity criteria outlined above (greater than 50 lines and less than 400 lines). These data were previously reported as a positive control in an experiment evaluating the effects of hypoxia on alarm cue response of fathead minnows [32].

Post-stimulus response data were analysed using ANCOVA in JMP Pro 15 software (type III sums of squares, 0.05 alpha level). Treatment type (control water or alarm cue) was treated as the categorical predictor, with the pre-stimulus behaviour as a covariate.

### (b) Histological examination

Twenty-nine Pahrump poolfish and seven fathead minnows were sacrificed using a lethal dosage of MS-222 (approx. 500 mg l^−1^), and a 3–4 mm section of skin was taken from the nape region [6,33]. Thin-sectioned histological samples were stained and mounted on slides and then digitally scanned using a MoticEasyScan Slide Scanner using Plan Apochromatic objective (20 × 0.75) with image detail equivalent to 40× lens. The number of visible club cells was counted for each slide and normalized using the estimated area of epithelial tissue to club cell density per mm² of skin using Image-Pro Premier. These data were used to estimate club cell density (club cells per mm^2^ of skin).

Data were analysed with JMP Pro 15 software. We used a likelihood chi square to test for inter-species differences in club cell prevalence. Due to small sample sizes, we used a permutation procedure [34] to test for differences in club cell density (club cells per mm^2^) where we performed a *t*-test to obtain the empirical difference in club cell densities between the two species and then conducted a permutation test. For each permutation, the observed club cell density estimates were randomized between the two species and the inter-species difference was calculated. This procedure was repeated 9999 times, along with the observed empirical difference, to create a distribution of 10 000 inter-species club cell differences expected by chance. The *p*-value was calculated as the proportion of random inter-species differences (absolute value of the difference between means) greater than or equal to the absolute observed difference, making it analogous to a two-tailed *t*-test.

## 3. Results

**Figure 1.**
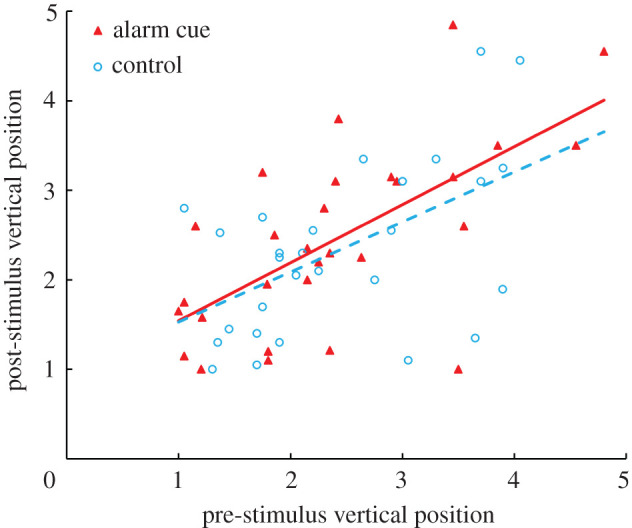
The mean vertical position (bottom as 1 and surface as 5) of Pahrump poolfish before and after the addition of alarm cue (solid triangles, solid line) or dechlorinated tap water as a control (open circles, dashed line) are shown. (Online version in colour.)

**Figure 2.**
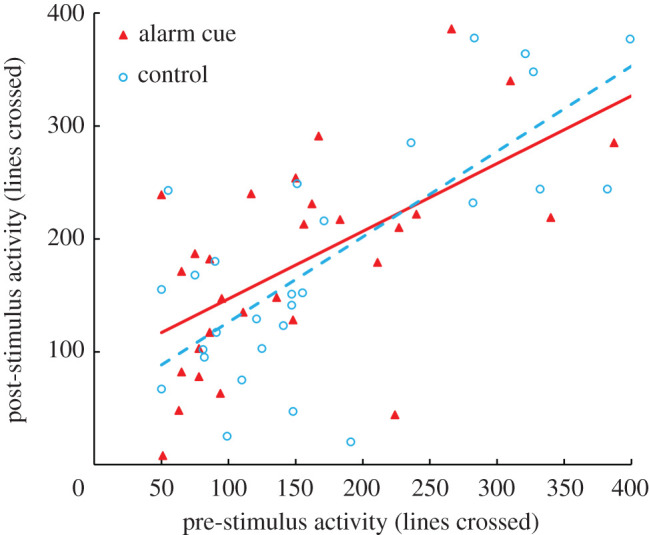
Activity measured as the number of lines crossed during a 5 min period is shown for Pahrump poolfish exposed to alarm cue (solid triangles, solid line) or dechlorinated tap water as a control (open circles, dashed line). (Online version in colour.)

Pahrump poolfish did not respond behaviourally to conspecific alarm cues. Poolfish post-stimulus position was not significantly affected by cue (*F*_1,54_ = 0.45, *p* = 0.505; figure 1). Further, the interaction between cue type and pre-stimulus position on post-stimulus position was not significant (*F*_1,54_ = 0.16, *p* = 0.694; figure 1), indicating that the covariance (i.e. slope) between pre-stimulus behaviour and post-stimulus behaviour did not differ between cue treatments. Similarly, post-stimulus Pahrump poolfish activity was not affected by cue type (*F*_1,54_ = 0.27, *p* = 0.607; figure 2). The interaction term was also not significant (*F*_1,54 =_ 0.63, *p* = 0.429; figure 2).

**Figure 3.**
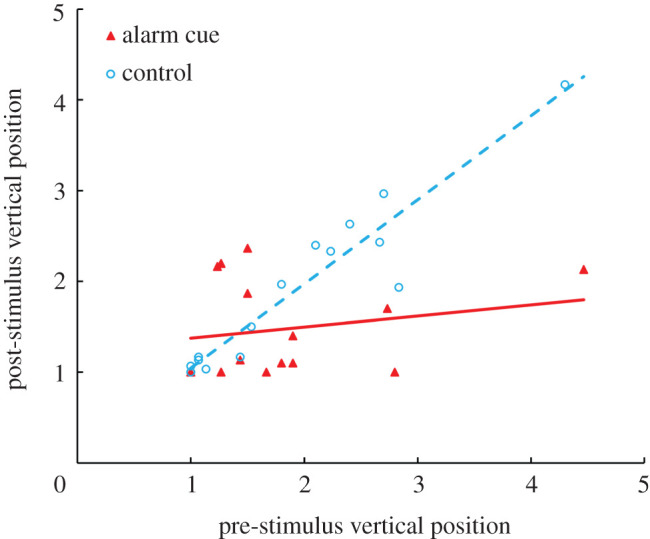
The mean vertical position (bottom as 1 and surface as 5) of fathead minnows before and after the addition of alarm cue (solid triangles, solid line) or dechlorinated tapwater as a control (open circles, dashed line) are shown. (Online version in colour.)

**Figure 4.**
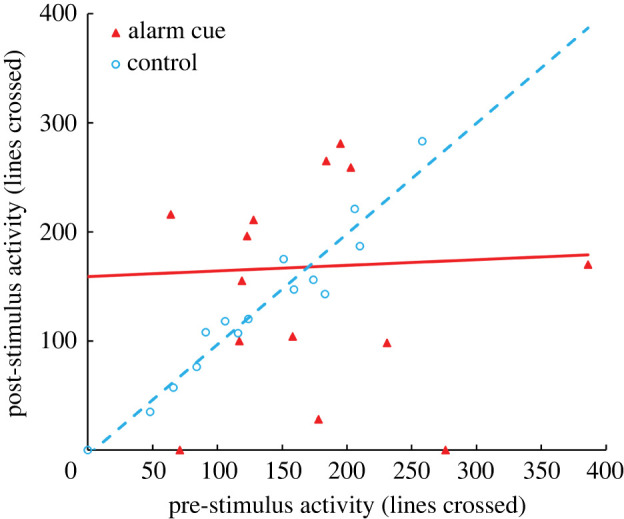
Activity measured as the number of lines crossed during a 5 min period is shown for fathead minnows exposed to alarm cue (solid triangles, solid line) or dechlorinated tap water as a control (open circles, dashed line). (Online version in colour.)

Fathead minnows, by contrast, showed strong behavioural responses to alarm cue. We observed a significant interaction between cue type and pre-stimulus behaviour for vertical distribution (*F_1,26_* = 19.685, *p* < 0.001; figure 3). Similarly, we observed a significant interaction between cue type and pre-stimulus activity (*F*_1,26_ = 4.686, *p* = 0.040; figure 4). Because of the significant interaction between pre-stimulus behaviour and cue type, we did not test for a treatment effect for post-stimulus vertical position or for post-stimulus activity.

**Figure 5.**
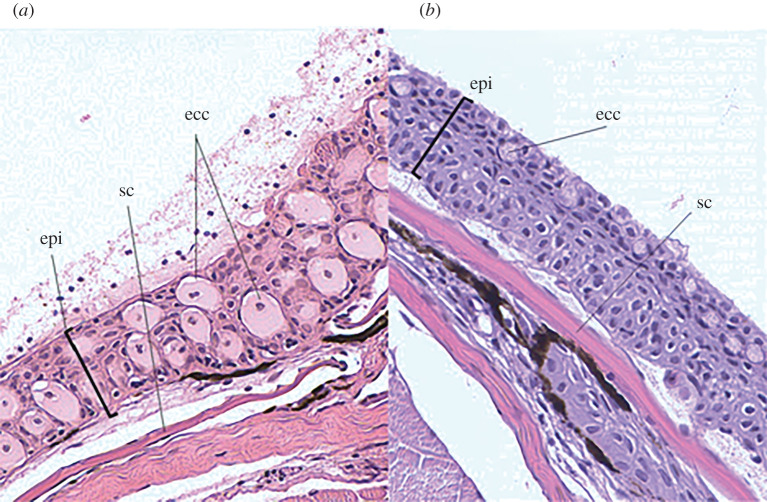
Digitized images of epithelial club cells for a fathead minnow (*a*) and Pahrump poolfish (*b*) are shown. Samples were stained, thin sectioned and then mounted on slides which were digitally scanned using a MoticEasyScan slide scanner with a Plan Apochromatic objective (20 × 0.75) with image detail equivalent to 40× lens. Epi, epidermal thickness; SC, scale; ECC, epidermal club cell. Club cells were classified as unstained, circular cells found within the epithelial tissue on scales and did not open to the surface of the tissue layer. (Online version in colour.)

Club cell prevalence differed between species (*χ*^2^ = 16.06, *p* < 0.001). Club cells were observed in 100% of fathead minnows (*n* = 7) and 24% of Pahrump poolfish (*n* = 29) (figure 5). Club cell densities were significantly higher for fathead minnows (1023.6 ± 86.3 per mm^2^; mean ± s.e.) compared to Pahrump poolfish (17.1 ± 8.5 per mm^2^; *p* < 0.001; figure 5).

## 4. Discussion

Pahrump poolfish did not respond behaviourally to conspecific alarm cues, which contrasted sharply with our positive control where fathead minnows showed strong responses to conspecific alarm cues. This contrast demonstrates that the lack of a response by Pahrump poolfish is not an experimental artefact of our experimental procedures. The behavioural responses may be partially explained by reduced alarm cue signal strength, as the epidermal club cell densities were much lower for Pahrump poolfish compared to the fathead minnows. It is also possible that reduced investment in club cells was accompanied by a reduced ability to detect cue.

The lack of anti-predator behaviour in Pahrump poolfish in response to conspecific injury-released chemical alarm cue is consistent with the predator naiveté hypothesis that has been widely reported for other insular populations [1–3]. Pahrump poolfish lack the ability to use olfactory cues of active predators and respond accordingly, which is noteworthy because virtually all small-bodied fishes (e.g. fathead minnows) exhibit anti-predator behavioural responses to conspecific alarm cues ([7], and citations therein). Concordantly, we found that fathead minnows had significant anti-predator responses to conspecific alarm cue.

Alarm cue represents the unconditioned stimulus in a powerful form of associative learning known as releaser-induced recognition learning [35]. Fishes rely upon releaser-induced recognition learning to acquire recognition of novel predators [7,36,37]. Without an innate response to conspecific alarm cues, Pahrump poolfish may be unable to acquire recognition and subsequently avoid invasive predators [7,13]. To our knowledge, Pahrump poolfish provide the first reported example of a species that lacks an olfactory-based chemical alarm cue system to facilitate the assessment of predation risk.

**Figure 6.**
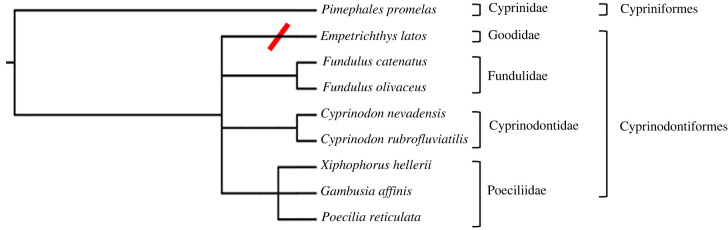
The phylogenetic relationships among species within the order Cyprinodontiformes are shown using the fathead minnow (Family Cyprinidae) as an outgroup. The diagonal (red) mark denotes the absence of alarm cue responses for Pahrump poolfish (*Empetrichthys latos*; this study), but alarm cue responses were observed for the other listed species including Amargosa pupfish (*Cyprinodon nevadensis amargosae*), Red River pupfish (*Cyprinodon rubrofluviatilis*), western mosquitofish (*Gambusia affinis*), green swordtail (*Xiphophorus hellerii*), guppy (*Poecilia reticulata*), northern studfish (*Fundulus catenatus*), black-spotted topminnow (*F. olivaceus*) [38–44] and fathead minnow [45] (also this study). The behavioural responses reported for northern studfish were in response to chondroitin sulfate, a presumptive chemical component of alarm cue substance.

Assessing the phylogenetic context of alarm cue presence/absence is challenging due to the limited sampling of this trait. To date, Pahrump poolfish is the only species within the family Goodeidae that has been evaluated for this trait. We evaluated the phylogenetic pattern of alarm cue by mapping this trait onto a tree that included Pahrump poolfish and seven additional species within the order Cyprinodontiformes (figure 6; phyloT tool based on NCBI Taxonomy [46]). This phylogeny infers secondary loss of alarm cue response in Pahrump poolfish, but a more comprehensive picture of the phylogenetic gains/losses of this trait await sampling of additional species within the Goodeidae family.

At a finer scale, the evolutionary history of alarm cue responses is difficult to assess as Pahrump poolfish is the only surviving taxon of its genus, *Empetrichthys*, due to the extinction of two other subspecies (*E. l. concavus* and *E. l. pahrump*) and a congeneric species (*E. merriami*) in the middle of the last century [16,47]. These four taxa were isolated from each other at the end of the Pleistocene, and thus the absence and/or presence of alarm cue responses cannot be determined among relatives of Pahrump poolfish within its own genus, subfamily or family.

We recognize a few caveats for interpreting our findings. For example, the low prevalence and low densities of club cells for Pahrump poolfish is notable because others have reported that club cells are a prominent component of epidermal tissue across many taxa [8]. We point out, however, that reduced predation pressure is not expected to reduce club cell abundance. The function of club cells has been widely debated, but one hypothesis is that club cells play an immune function against pathogens [6,8]. Thus, it is possible that evolution in isolated habitats may also reduce pathogen pressure, but this hypothesis remains to be critically evaluated. Even though club cell densities were low for Pahrump poolfish, it is worth noting that fathead minnows can exhibit anti-predator behaviour when presented with skin extract lacking club cells suggesting the presence of other chemical cues in the epidermis exclusive of club cells [48].

One additional possibility is that a few generations in captivity have resulted in the absence of alarm cue recognition due to phenotypic plasticity or contemporary evolution (*sensu* [49]). Such a finding would be of equal interest to conservation biologists largely because captive stocks are often used for restoring field populations. However, we note behavioural responses to alarm cues by captive stocks of laboratory species such as fathead minnows and zebrafish are widely reported [7].

It is important to note that our findings are consistent with historic impacts of invasive species on Pahrump poolfish populations [22]. We hypothesize that a lack of behavioural response to chemical alarm cue, and therefore an inability to recognize novel predators, may have contributed to the decline of Pahrump poolfish at Spring Mountain Ranch in 2016, where the Pahrump poolfish population plummeted from over 12 000 fish to less than 1000 fish following the sequential introduction of red swamp crayfish in 2012 and western mosquitofish in 2015 (Kevin Guadalupe, Nevada Department of Wildlife, pers. comm.). Further, Miller *et al*. [16] suggested that invasive red swamp crayfish contributed to the extinction of the closely related Ash Meadows poolfish (*E. merriami*). In addition, red swamp crayfish severely reduced the survival of adult Pahrump poolfish in experimental mesocosms [23]. Finally, experimental Pahrump poolfish populations failed to produce juveniles in the presence of non-native western mosquitofish [22,23].

While broadly consistent with the predator naiveté hypothesis, our findings raise several interesting questions. For instance, predator naiveté often involves failure of prey to recognize novel predators [50], whereas the loss of chemosensory risk assessment is a more generalized response to predator risk. However, the native habitat of Pahrump poolfish most likely included invertebrate predators such as dragonfly naiads, which also inhabit nearby desert aquatic systems [51] and have been reported to prey on larval fish [52]. Therefore, it is possible that Pahrump poolfish have retained other anti-predator behaviours to reduce such risk.

In conclusion, our results suggest that Pahrump poolfish have lost the ability to respond to chemical cues released immediately following a predation event, and by extension they have lost the ability to acquire recognition of novel predators. These findings, combined with the vulnerability of poolfish populations to repeated impacts of non-native predators, suggest that Pahrump poolfish should continue to be managed in single-species refuge habitats [22]. In the absence of non-native predators, Pahrump poolfish populations have flourished as evidenced by the repeated success of establishing (and re-establishing) single-species refuge populations [22,26]. Thus, continued vigilance for detecting the introduction of non-native species will be necessary to ensure the persistence of Pahrump poolfish.

## Data Availability

Behavioural and histological data are publicly available in the Dryad Digital Repository (doi:10.5061/dryad.z8w9ghxdt) [31]. The data are provided in the electronic supplementary material [53].
